# Supplementary figures and images for: Foveal neurons of the monkey superior colliculus signal trans-saccadic prediction errors
Source: PLoS Biol. 2025 Jun 23;23(6):e3003246. doi: 10.1371/journal.pbio.3003246 (PMC12212877; doi:10.1371/journal.pbio.3003246)

**a**

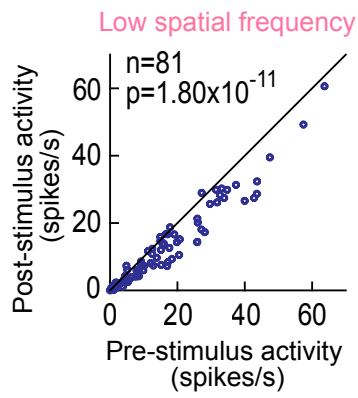

**b**

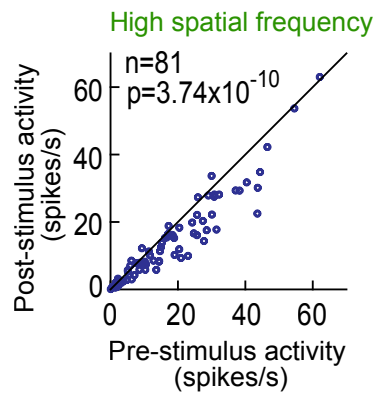

Supplement: S1 Fig — (a) For all neurons included in our main analyses of Figs 1–4 and 7–9, we measured neural activity 50–150 ms after extrafoveal stimulus onset (during maintained gaze fixation), and we compared it to pre-stimulus activity. Here, the appearing stimulus was a circular patch containing a low spatial frequency texture. There was a reduction in activity, rather than visual bursts. (b) Similar observations for the case in which the extrafoveal stimulus had a high spatial frequency texture embedded within it. The figure’s underlying data are included in S10 Data. (PDF) [file pbio.3003246.s001.pdf]

**a**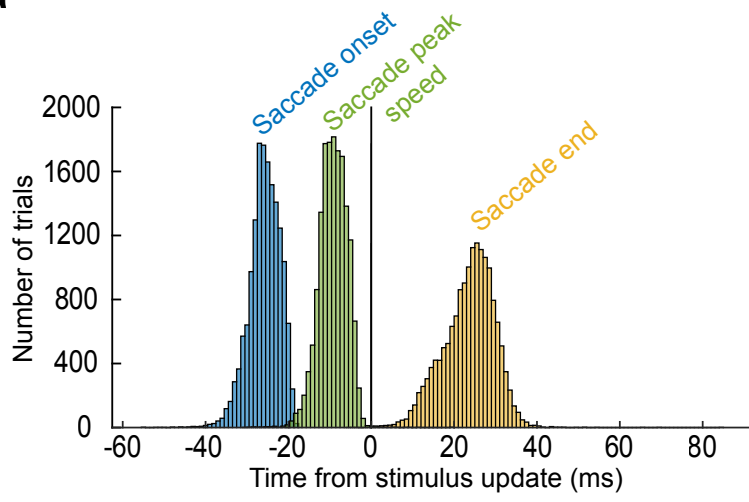**b**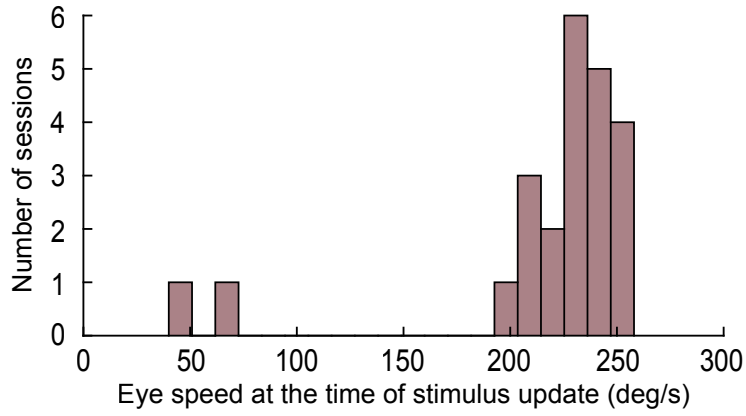**c**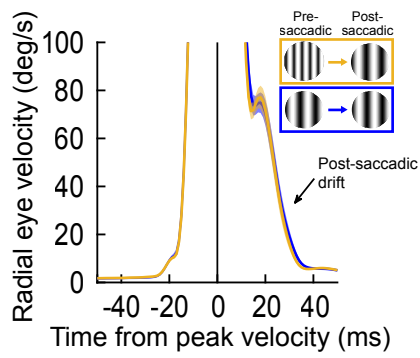**d**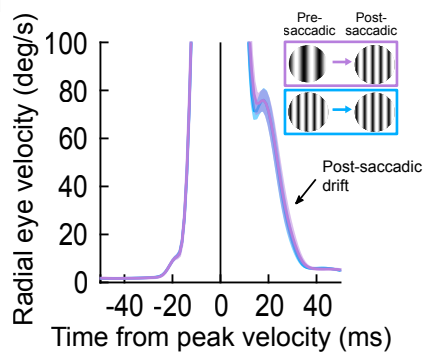**e**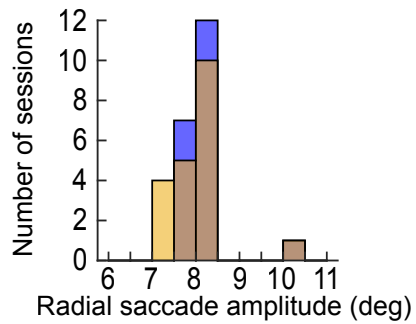**f**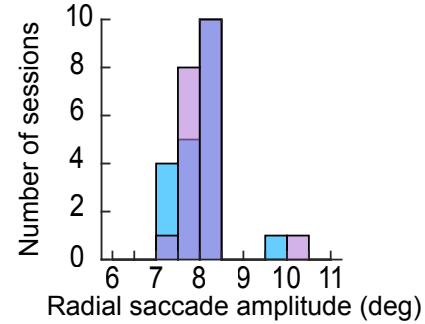

Supplement: S2 Fig — (a) For all trials containing a real or fictive image update (from the basic paradigm of spatial frequency changes and circular outline shapes of the saccade targets), we measured (also across all sessions) the times of saccade onset, saccade peak velocity, and saccade end relative to when the image flip event was completed. We always had intra-saccadic image updates in our experiments. (b) Importantly, the eye speed was always high by the end of the image update. Here, for each session, we calculated the average eye speed by the end of the image update event, and we plotted a histogram across sessions. For the great majority of sessions, the image speeds on the retina by the end of intra-saccadic image updates were >150 deg/s. For the two sessions with speeds of approximately 50 deg/s, the saccade targets were placed a bit closer to the fovea (thus having smaller saccades) because the sessions involved other experiments involving the primary visual cortex (we thus tailored the target locations to the cortical neurons explored during the same sessions for other purposes). However, eye speed was still relatively large during these outlier sessions. (c, d) For the main conditions of Figs 2 and 3 we plotted radial eye velocity on control and intra-saccadic stimulus change trials. Each plot shows the average velocity curve across sessions (with horizontal saccade target locations, which were the majority), and the error bars show SEM across sessions. The saccadic profiles were not affected by intra-saccadic display updates, including post-saccadic drift speeds. This means that the saccades were not truncated by the stimulus changes. Similar observations were made for the few oblique sessions. (e, f) Similar to c, d but now plotting radial saccade amplitude across sessions. There was again no evidence of saccade truncation by the intra-saccadic stimulus changes. Similar conclusions were reached for all other experiments (e.g., with shape changes). The figure’s underlying [file pbio.3003246.s002.pdf]

**a**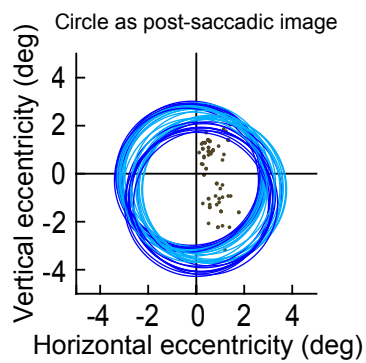**b**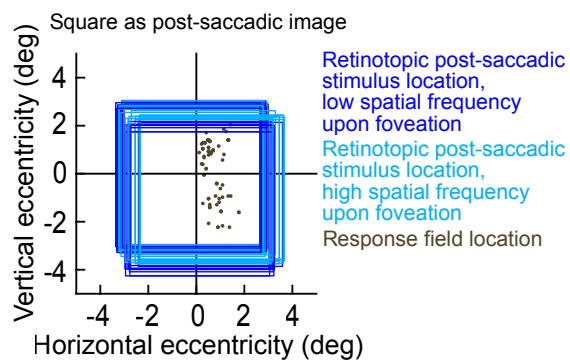

Supplement: S3 Fig — (a) Across trials and sessions from our basic paradigm, we plotted the retinotopic position of the saccade-target patch (circles) upon saccade end. There was variability due to variability in saccade metrics. However, the foveated image patches were always encompassing our recorded foveal neurons (dark gray spots indicating the foveal RF hotspot locations). Thus, we always had robust visual reafferent responses. Note also that such jitter in saccade landing should have weakened reafferent responses in some cases, rather than strengthened them, because it could cause visual stimulation at sub-optimal RF positions. This suggests that our main results (dominated by elevated reafferent responses) are not explained by jitter in saccade landing positions. (b) Similar observations from the shape-change trials (Methods) of our paradigm. Here, the foveated stimulus had a square outline instead of a circular one. The figure’s underlying data are included in S12 Data. (PDF) [file pbio.3003246.s003.pdf]

**a**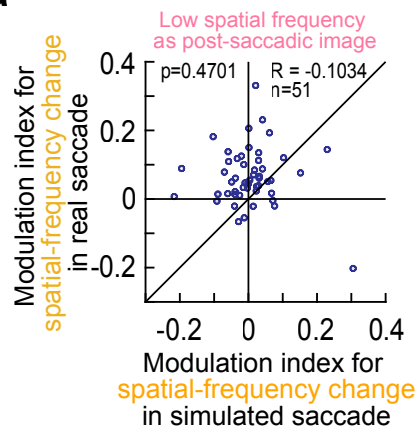**b**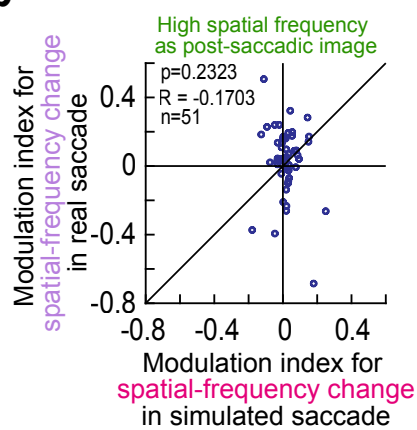

Supplement: S4 Fig — (a) For a low spatial frequency foveated patch after either real or simulated saccades, we plotted the modulation indices from Fig 6g on the x-axis (for simulated saccades) and those from Fig 3g on the y-axis (for real saccades). The shown neurons are those for which we ran both conditions together in the same session. In the simulated saccade case (x-axis), the modulation indices straddled zero; however, in the real saccade case (y-axis), the modulation indices were largely positive. Thus, there was no correlation between the two situations (p = 0.4701). (b) Similar observations for a high spatial frequency foveated image (p = 0.2323). The figure’s underlying data are included in S13 Data. (PDF) [file pbio.3003246.s004.pdf]

**a**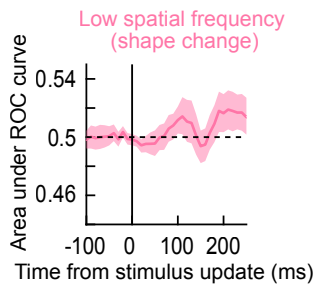**b**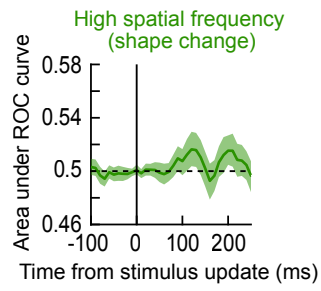**c**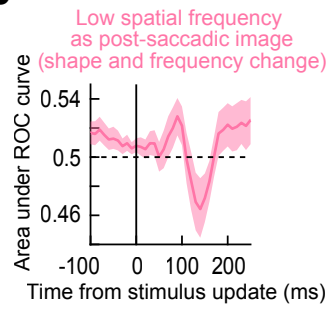**d**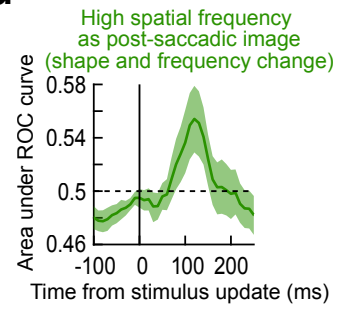**e**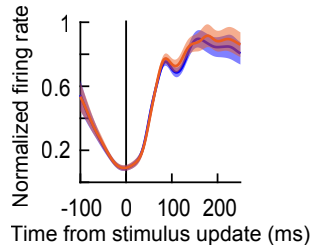**f**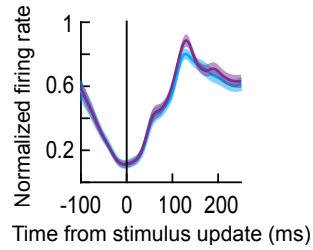**g**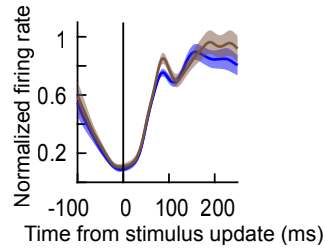**h**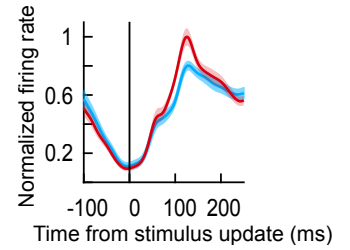

Supplement: S5 Fig — (a, b) ROC analyses for the experiments of Fig 7. Whether landing on a low or high spatial frequency texture, there was an elevation in area under the ROC curve in the reafferent response epoch. (c, d) The effects were larger for the combined shape and spatial frequency change trials (Fig 8). (e, f) Population firing rate dynamics for the experiments of Fig 7. (g, h) Population firing rate dynamics for the experiments of Fig 8. Error bars denote 95% confidence intervals for a–d and SEM for e–h. The figure’s underlying data are included in S14 Data. (PDF) [file pbio.3003246.s005.pdf]

**a**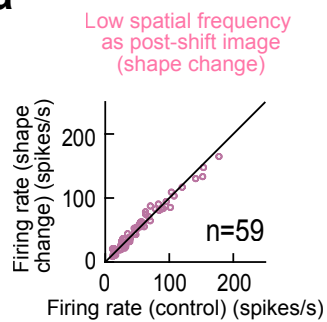**b**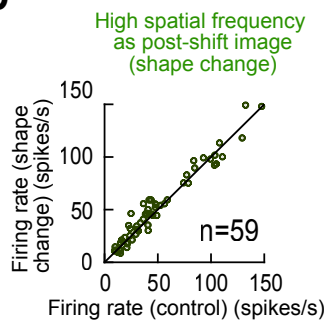**c**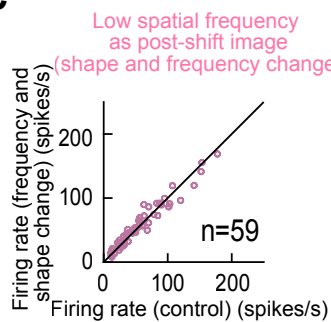**d**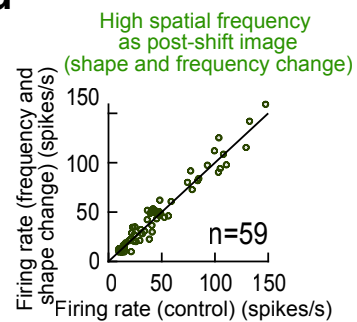**e**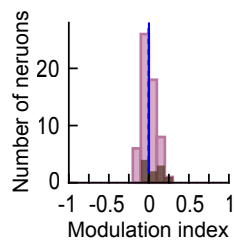**f**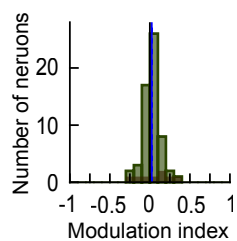**g**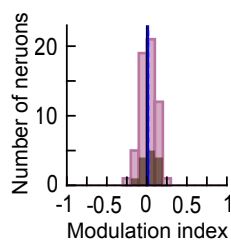**h**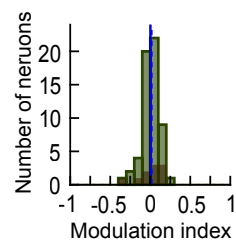

Supplement: S6 Fig — (a, b) Population results like in Fig 6e and 6f, but now for the shape change trials and a low (a) or high (b) spatial frequency post-translation texture. There were weaker differences in visual response strength relative to control than with real saccades. (c, d) Similar observations for the shape plus spatial frequency change trials. (e, f) Neural modulation indices for a, b (p = 0.8 and 0.049 for e and f, respectively; Wilcoxon signed-rank test). (g, h) Neural modulation indices for c, d (p = 0.22 and 0.40 for g and h, respectively; Wilcoxon signed-rank test). The figure’s underlying data are included in S15 Data. (PDF) [file pbio.3003246.s006.pdf]

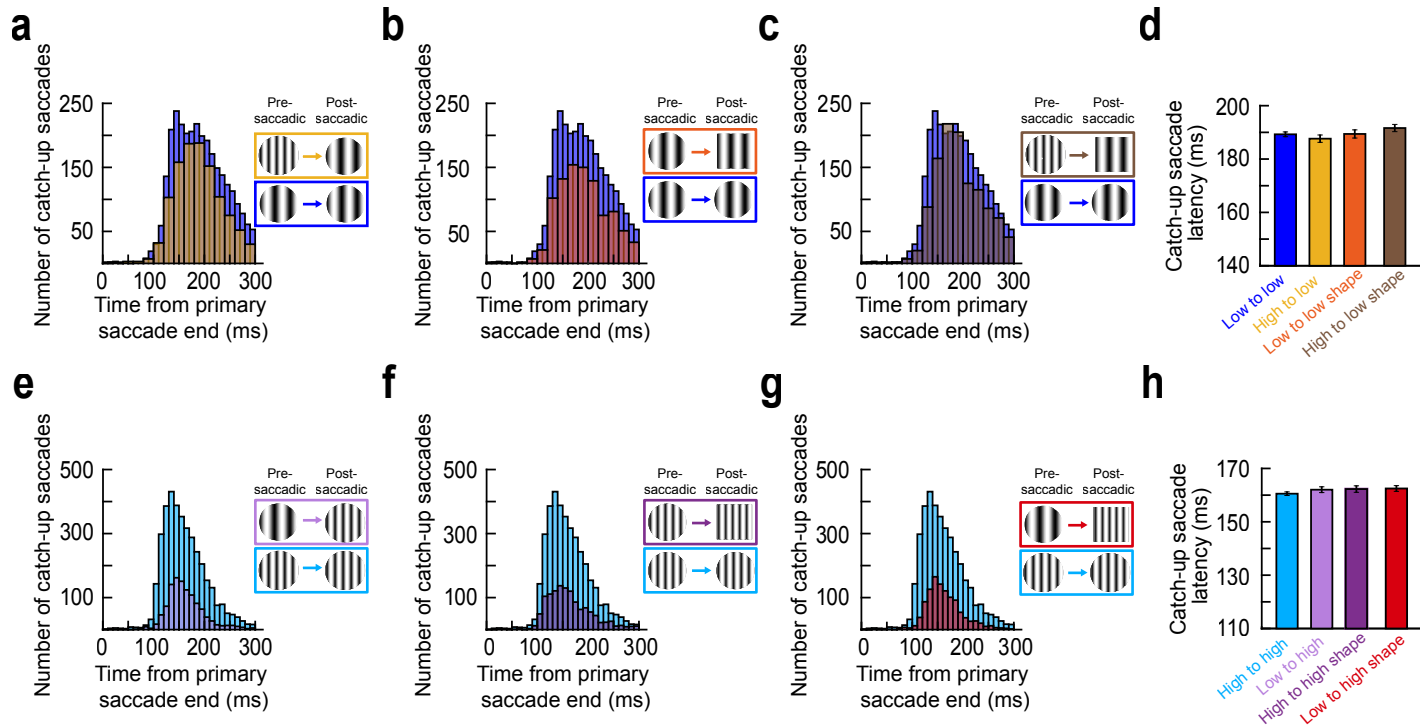

Supplement: S7 Fig — (a) For the basic experiments of Figs 2 and 3, and when landing on a low spatial frequency texture, the distribution of catch-up saccade times (for the first saccade after foveating the target) was very slightly skewed towards longer latencies on intra-saccadic change trials, but there were also saccades with very short latency. (b, c) Similar observations for the shape (b) and shape plus frequency change experiments (c). (d) The averages of the distributions in a–c. For the spatial frequency condition (High to low), the average latency was reduced relative to control, likely due to the few saccades with very express latencies. With shape plus frequency changes, it was increased again, but there was (overall) no significant effect of condition on catch-up saccade latency. A Kruskal-Wallis nonparametric ANOVA across all conditions showed no significance (p = 0.2617). (e–h) Same as a–d but when landing on a high spatial frequency texture. Here, the catch-up saccade latencies were generally faster than when landing on a low spatial frequency (compare h to d). Perhaps as a result, it was easier to see the influence of intra-saccadic stimulus changes. Specifically, catch-up saccade latencies increased relative to control (High to high), and there was a significant effect across conditions in h (p = 0.0049). Thus, elevation of foveal SC activity on intra-saccadic change trials was associated with slightly delayed corrective saccades. This is an expected consequence of prediction error signaling and also of elevated foveal SC activity. Error bars denote SEM. Note that the histograms show fewer numbers of catch-up saccades on intra-saccadic change trials when compared to control trials. This is because there were fewer intra-saccadic change trials than control trials in our experiments (Methods). The figure’s underlying data are included in S16 Data (the summary statistics in d, h are those obtained from the raw distributions in the other panels). (PDF) [file pbio.3003246.s007.pdf]
