# Supplementary material for: Foveal neurons of the monkey superior colliculus signal trans-saccadic prediction errors
Source: PLoS Biol. 2025 Jun 23;23(6):e3003246. doi: 10.1371/journal.pbio.3003246 (PMC12212877; doi:10.1371/journal.pbio.3003246)
Supplement: Attachment — Submitted filename: Zhang_RESPONSES.docx [file pbio.3003246.s026.docx]

**Responses to Reviewer Comments on:**

**“Signaling of trans-saccadic prediction error by foveal**

**neurons of the monkey superior colliculus”**

**PBIOLOGY-D-25-00819R1**

**May 25, 2025**

We thank the editors and all three reviewers very much for the opportunity to publish our work in this journal.

We have now revised the manuscript in accordance with all of the reviewer recommendations. We have also formatted the paper according to all of the journal’s instructions.

Below, we provide detailed responses to all of the reviewer comments.

We sincerely hope that the paper is now ready for publication.

**Responses to Reviewer 1**

The study of Zhang et al. uncovers an interesting line of evidence supporting the role of primate superior colliculus in detecting a change in the visual field during a saccadic eye movement. By using a simple paradigm in which a static visual image undergoes a change contingent upon a saccade start, they show that the neurons in primate SC can encode that change regardless of these visual neurons' own tuning preference for stimulus features. They also demonstrate that the magnitude of this change detection in SC neurons scales with the amount of change in the visual stimulus in shape and spatial frequency. Overall, they report a general trend in the peri-saccadic activity of SC neurons consistent with a mechanism of detecting prediction error. 
The paper overall is excellent. The questions are well-defined, the results are clear, and the statistical methods are sound. The paper is also clearly written. This study addresses an important problem in peri-saccadic visual perception - how does the visual system maintain stability and detect change across saccades? This question has somewhat gone out of fashion in recent neuroscience work because of some controversial findings in the literature. Zhang et al's study offers evidence for a refreshing alternative to approaching the problem. Therefore, this study is a significant contribution to the field. 
I don't have any major issues with the claims and results. If the authors could address the following minor comments, I believe that would improve the paper.

OUR RESPONSE: Thank you very much for the support and encouragement. We have made a genuine effort to address all of your and the other reviewers’ comments.

1.	A. The introduction gives the example of the Mona Lisa to motivate the visual stability problem. However, I found the example a bit confusing. My confusion was that when I look at Mona Lisa's eyes and saccade to her mouth, I don't perceive a stable portrait. I perceive that her smile suddenly vanishes. This 'fleeting smile' is what made this artwork so popular. Margaret Livingstone has published a paper explaining this phenomenon (<https://doi.org/10.1126/science.290.5495.1299b>). The visual system detects a change in the portrait, which is not actually there in real-time. It is simply a function of how visual receptive fields are arranged topographically - low spatial frequency-tuned neurons tile more of peripheral vision than foveal. Maybe the authors can use a different example that doesn't cause this particular confusion. 

OUR RESPONSE: We apologize for this. We agree that this example was probably not the most ideal one to use here. We have now rewritten this example (please see **lines 60-63** of the revised manuscript). As suggested in a later comment, we have also revisited the Mona Lisa example in the Discussion section, this time from the perspective of spatial vision in the periphery and fovea (please see below for more details).

B. Conceptually, the framing of the authors' main question needs to explicate certain nuances. In line 638, they say - "our focus here: how is visual sensation of a stably present visual object in the environment handled when an eye movement brings this object inside a given neuron's RF?". Due to the gradient of spatial frequency over a visual retinotopic map, a peripheral object, otherwise stable, will necessarily look different to visual neurons at the fovea. How does the visual system deal with the mismatch of spatial frequency of its filters? I understand that the authors are not addressing this question in their study, but this nuance could be explicitly mentioned to make the discussion richer and avoid confusion. 

OUR RESPONSE: Thank you. We have now added text to the Discussion section addressing this point, and also linking it to the Mona Lisa. Please see **lines 703-723** of the revised manuscript. Briefly, we believe that our observations of scaling of prediction error signaling with the magnitude of the intra-saccadic feature changes provides interesting hints on this matter. Details are provided in the manuscript.

2.	Along the same lines, maybe the authors can discuss if the visual system uses a similar mechanism to detect a peri-saccadic perceptual change not because of a physical change in the visual field but because of the spatial frequency gradient on the visual images - exactly like the 'fleeting smile' on Mona Lisa. Given the recent findings of short latency responses to faces in primate SC (<https://doi.org/10.1016/j.neuron.2024.06.005>), I think it is a great opportunity for the authors to link their findings of prediction error in SC to short latency face detection in primate SC. This could be one way to use the same example but more powerfully and less confusingly. But I leave it up to the authors to decide. 

OUR RESPONSE: Yes, as mentioned above, we have now added information to the Discussion section that is relevant for this point and the intriguing object and face visual responses in the SC. Please see **lines 703-723; 725-732** of the revised manuscript.

3.	The methodology would be more robust if the authors could provide, if possible, a photodiode readout of the frame luminance across time. This is to be absolutely sure that the saccade-triggered change in visual stimulus occurred mid-flight. If the current calculations (I assume based on software and hardware TTLs) could be corroborated with actual events on the screen, all doubts about instrumentation delays would be cleared. 

OUR RESPONSE: We have now clarified this information further in Methods (please see **lines 851-862** of the revised manuscript).

Briefly, in our setup, we had direct access to the vertical blanking signal of the graphics card. Thus, we synchronized our system to every frame of the display. In the earlier days of the Hafed laboratory (e.g. Chen & Hafed, J. Neuroscience, 2013), we also conducted very extensive tests on timing by combining the above-described system with direct photodiode measurements. We are confident that our timing of intra-saccadic changes was successful.

Consistent with this, all of our reafferent responses were sharply aligned to the saccades, whether we had an intra-saccadic feature change or not. This was the case not just in the example neurons (e.g. Figs. 1, 2), but also in the new population firing rate dynamic plots that we have added in the revised manuscript in response to additional comments below (e.g. Fig. 3a, b).

**Responses to Reviewer 2**

The authors identify a potential mismatch signal between expected and experienced spatial frequencies across saccades in foveal neurons of the macaque superior colliculus. In particular, reafferent visual firing rates of foveal SC neurons are slightly higher when the stimulus that is foveated has its spatial frequency changed unexpectedly during a saccade. They find that this phenomenon is unique to the condition where the animal initiates a saccade, and scales with the number of features that are mismatched pre- and post-saccadically (i.e., firing rates for shape + spatial frequency changes are higher than for spatial frequency alone). 

Comments

Since one main goal of making saccades is to foveate targets of interest, this work showing that unexpected events across saccades are processed at the center of gaze is a valuable addition to the literature on the neural basis of visual stability across saccades. 

OUR RESPONSE: Thank you very much for carefully reviewing our manuscript. We have now addressed all reviewer comments in the revised manuscript.

1)	My biggest concern is that the main mismatch effects shown across the population of neurons in Figs. 3, 6, and 7 are small, and I'd want to be sure that they are robust across potential analysis choices. For example, for all the population visual response comparisons, the authors calculate reafferent firing rates aligned to the change event in all conditions including those with a saccade. However, this period includes the offset of the saccade when the eyes are still in flight and a rebound in the neuronal activity after a period of saccadic suppression, both of which may introduce spurious transients in the visual responses. Are the same effects observed even when the reafferent period is aligned to the offset of the saccade rather than the visual change? 

OUR RESPONSE: Thank you for raising this important and well-taken point.

We are very confident that our results are robust to our choice to align on the stimulus-change event, and we have taken the following measures in the revised manuscript to convince readers of this:

- We have clarified that we have repeated all of our statistical analyses when aligning all data to saccade end rather than to stimulus update time (please see **lines 971-981**). Doing this confirmed that we could reach the very same conclusions throughout the manuscript. In our mind, this is expected since stimulus updates occurred when eye speed was still relatively high (e.g. Fig. 2a and S2b Figure), and since they also occurred during maximal saccadic suppression (e.g. Figs. 2b, c, 3a, b). Indeed, aligning to saccade end only introduced a constant shift in the firing rate curves, confirming the repeatability of our online saccade detection approach. Our primary reason for using the stimulus-change event as the alignment time point was to also use stimulus-change events for alignment when no saccades happened (i.e. in the simulated saccade conditions; e.g. please see **lines 980-981**)
- We have clarified that when we aligned to stimulus-change events, we also always did this for “fictive” changes in the control conditions. Thus, any small jitter in online saccade detection applied to both control and stimulus-change trials (e.g. please see **lines 202-203; 228-231**)
- We have added new population firing rate curves for all analyses, not just example neurons and summary statistics (e.g. Figs. 3a,b, 6a, b, and S5e-h Figure), as well as time-varying ROC analyses (e.g. Figs. 3c, d, 6c, d, and S5a-d Figure). This was important because it demonstrated that the relatively small effects still held across the population, and were not “washed” out due to too much variability in online saccade detection across different sessions and neurons
- We have confirmed that reafferent responses in control trials had similar timing to those in the stimulus-change trials, again suggesting systematic and repeatable online saccade detection (and therefore proper alignment to a specific time point within the saccades; whether this time point is the endpoint or not is irrelevant and just introduces a time shift). For example, please see the raw data in Figs. 1d, 2b, c (e.g. please see **lines 228-231**)
- We have clarified that we have measured the eye speed at the time of stimulus change to confirm that it occurred during maximal saccadic suppression (e.g. S2b Figure) (e.g. please see **lines 249-253**)
- Consistent with this, we have added new analyses of eye speed (S2c, d Figure) and saccade radial amplitude (S2e, f Figure) demonstrating that saccades were not truncated or otherwise altered by intra-saccadic stimulus changes. This means that we had very similar alignment to the saccade kinematics in the control and stimulus-change trials for the firing rate measures

2)	The main analyses show the mean firing rates and their modulation across conditions for individual neurons and a statistical comparison of those means across the population. It would also be informative to see what proportion of individual neurons show a significant difference between the change and control conditions across trials. 

OUR RESPONSE: We have now added the individually significant neurons to all histograms of modulation indices throughout the whole manuscript. Also, we reported the statistics for the individual example neurons described in the text.

3)	Were there any systematic differences in eye position or velocity, either as post-saccadic fixational eye movements/drift or during the instructed saccade, when the visual feature changed intrasaccadically? 

OUR RESPONSE: We have now added three new analyses addressing this point.

First, we plotted radial eye velocity, including post-saccadic drift epochs. Please see S2c, d Figure. This analysis revealed that saccades were not truncated or otherwise modified by the intra-saccadic stimulus changes. This is good to know because it supports our view that the neural effects were not artifacts of altering the saccades themselves.

Second, we also plotted radial eye displacement. Please see S2e, f Figure. Again, this analysis revealed that saccades were not truncated by the intra-saccadic stimulus changes.

Finally, we have now measured the timing of the first catch-up saccade after the primary saccade. Please see S7 Figure. Here, we saw elevated catch-up saccade latencies on mismatch trials, especially when landing on a high spatial frequency texture. For the low spatial frequency texture, the trends were similar, but there were few “express” catch-up saccades that affected the means. These observations are interesting because they support our conclusion that foveal SC activity was elevated on mismatch trials. When foveal SC activity is elevated, it is known that saccade generation might be rendered slightly harder due to competition of multiple motor goal representations (e.g. Hafed et al., 2008).

4)	Finally, in the Discussion section, the authors speculate on ways in which foveal neurons may have access to the pre-saccadic visual information in order to identify the difference between pre- and post-saccadic stimuli. These include neurons with larger receptive fields and visual information carried by motor neurons. The most straightforward possibility in my opinion is that of pre-saccadic remapping, which the authors mention but reject as a possible explanation. I did not follow the reasoning for why this is a conceptually unrelated phenomenon. If a visually responsive cell could, via remapping, briefly sample the part of the visual field that it will occupy after a saccade, would that not give it direct access to both the pre- and post-saccadic visual stimulus? However, this is only a relatively minor point of interpretation that does not change the presentation of the main observations here. 

OUR RESPONSE: We sincerely apologize for this. We did not mean to discount remapping as a potential contributor. Our wording was a bit unfortunate (sorry), but we were trying to delineate between spatial location representations and feature representations. Of course, we do acknowledge that spatial remapping also involves feature remapping, and we have made this clearer now. We have also been much more careful in the text description of remapping mechanisms. Apologies once again. Please see **lines 703-723; 734-741**.

**Responses to Reviewer 3**

How our visual system keeps a stable representation of the visual scene during saccadic eye movements is a crucial piece of the puzzle of how in a dynamically changing world our brain constructs a stable mental model resilient to changes in the flow of visual signals and other interruptions. In this study Zhang, Bogadhi and Hafed study how fovea representing neurons within the superior colliculus (SC) are modulated by information presented peripherally, when that information is going to be brought up to the fovea during an upcoming eye movement. It should be noted that, in spite of the significance of the fovea, in general in visual system electrophysiology experiments, the foveal representation is historically understudied and this also enhances the significance of the current paper. The manuscript is very well-written and the research questions are significant and properly studied. I did not find any major issues with the experimental design, analysis or
interpretation. Thus, there are only a few minor comments as listed below. However, I have a few comments, more reflecting of missed opportunities rather than necessary analysis, and I totally leave it to the authors to decide if they want to address them in this paper or leave it to be addressed in future manuscripts. 

OUR RESPONSE: Thank you very much for the encouragement and support. We have tried our best to address all of the reviewer comments.

Comment1: As pointed out by the authors, the transfer of information across various representations has been reported in several contexts. In spite the rich literature, there are not many studies investigating the neural mechanisms involved in such "rerouting of information". It would be great to know the authors' perspective on the neural machinery involved in such rerouting of information in the discussion section. Also, if their dataset allows, investigating how propagation of oscillatory activity might be coupled with information rerouting (e.g. similar to Neupane, Guitton, Pack PNAS 2017) could be a very good extension of the current study. Again, leaving it for the authors to decide when and how to dig deeper into the neural mechanisms involved.

OUR RESPONSE: This is a good point. We have now added text relating cortical oscillations and waves to potential SC mechanisms. Please see **lines 734-741**. We are currently working on a follow-up project that investigates the transition from sensory processing to motor generation in the SC, and we think that this other project would give us a much better chance to go deeper into such potential mechanisms. We hope to have the manuscript completed within the next few months.

Comment 2) Coding multiple locations can also be interpreted as having "mixed selectivity". Population-level analyses such as PCA have been shown to reveal hidden aspects of the neural code where single neuron analysis is missing to trace them. Even non-simultaneously recorded neurons can be used to trace how much information at the population level does exist regarding a peripheral target and how this information changes around the time of saccade. Again, I would leave it to the authors to decide whether they want to employ population-level analysis for this manuscript or not. 

OUR RESPONSE: We have added population firing rate dynamics and ROC analyses to the current revised manuscript (see below). However, even though we did perform the PCA analyses, we have elected to skip these analyses for the current manuscript. They did reveal expected modulations on the mismatch trials when compared to the control trials, but we felt that such analyses (or related ones) would be much more suitable for the follow-up study mentioned above, which explicitly explores the transformation from sensory processing to motor generation in the SC across all epochs (from stimulus onset, to go-signal, to motor execution). We feel that the current manuscript might lose too much focus if we made it even longer than it already is.

Comment 3) I was hopeful to see a figure depicting the tiling of the receptive fields around the fovea, or at least their centers. 

OUR RESPONSE: We did show the RF centers in S3 Figure. However, the color was probably too faint, and the mentioning of them in the text was not clear enough. We apologize for this. We have now clarified the reference to this figure in **line 253** in the main text. Moreover, we now show the RF centers with a higher contrast color in the actual figure (S3 Figure).

Comment 4) It seems imperative to show the normalized response of population of neurons (equivalent of figure 2b, c but for the population) before jumping to scatter plot-level description in figure 3. In general, the temporal dynamics at the population level is missing for almost all reported phenomena and it would increase the value of the paper to bridge the single neuron temporal response and population-level histograms/scatter plots with a population-level normalized response; and even preferably dynamics of an index such as ROC or modulation index across time. 

OUR RESPONSE: We fully agree that there was probably too big of a jump from example neurons to scatter plots in the original manuscript. Therefore, as suggested, we have now added normalized population responses for all relevant analyses (e.g. Figs. 3, 6, and S5 Figure). Similarly, we have also added ROC time courses for all relevant analyses, exactly as suggested.

Minor comments: 
-	Isn't it better to call it e.g. psuedosaccade in fig. 5? Calling it saccade might be confusing.

OUR RESPONSE: Yes, we now see that we had the legend labels as “pre-saccadic” and “post-saccadic”, which is indeed confusing. We fully agree. So, now, we have changed this to “pre-shift” and “post-shift”. We have also done this in Fig. 6 (we decided to split the original Fig. 5 into two separate figures given the addition of population firing rates and ROC analyses). Please see the new figures.

-	Putting p values in the figures right above where the sample sizes are reported (e.g. n=59) could help the reader grasp the significance of the figures quicker.

OUR RESPONSE: We agree, and we have now added them to all of the relevant figures. Thank you.
